# Supplementary material for: Systematic analysis of the glucose-PTS in Streptococcus sanguinis highlighted its importance in central metabolism and bacterial fitness
Source: Appl Environ Microbiol. 2024 Nov 25;91(1):e01935-24. doi: 10.1128/aem.01935-24 (PMC11784104; doi:10.1128/aem.01935-24)
Supplement: Supplemental material — Figures S1 to S12; Tables S1 to S4. [file aem.01935-24-s0001.pdf]

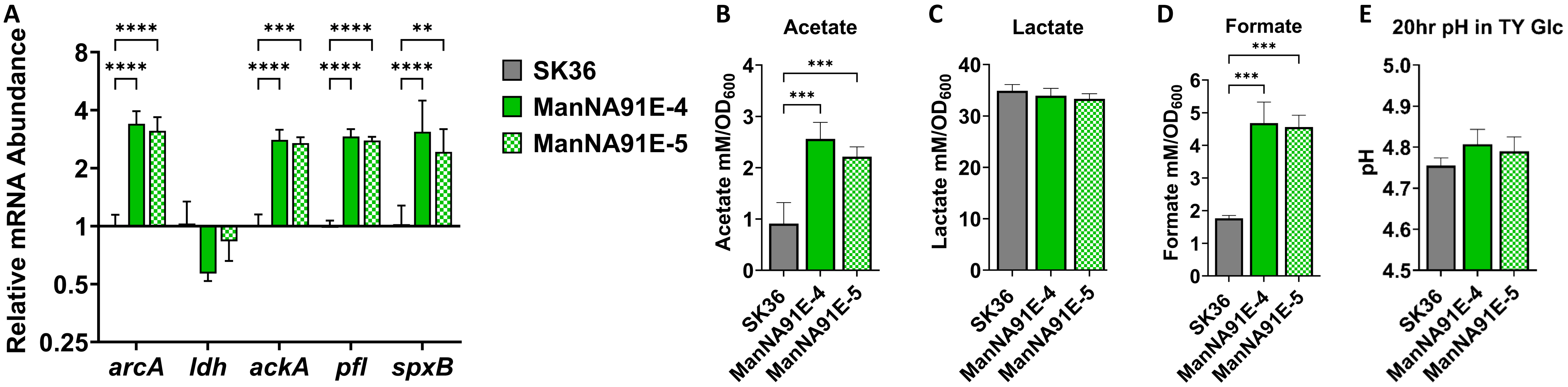

Fig S1 Characterization of two ManNA91E isolates. SK36 and its mutant derivative were grown to exponential phase (A-D) or for 20 hours (E) in TY (A, E) or TV (B-D) supplemented with 20 mM of glucose. (A) To measure abundance of mRNA levels of metabolic genes, RNA was extracted from cells and relative abundance was calculated relative to an internal control (*gyrA*). Cells grown in TV (B-D) had supernatants collected and were used to measure acetate (B), lactate (C), and formate (D). Cells grown for 20 hours had pH measured (E). Results are each an average of at least three biological replicates, with error bars denoting standard deviations. Asterisks represent statistical significance compared to the wild type according to two-way ANOVA (A) or a students' T-test (B-E) or (*, $P < 0.05$; **, $P < 0.01$; ***, $P < 0.001$; ****, $P < 0.0001$)

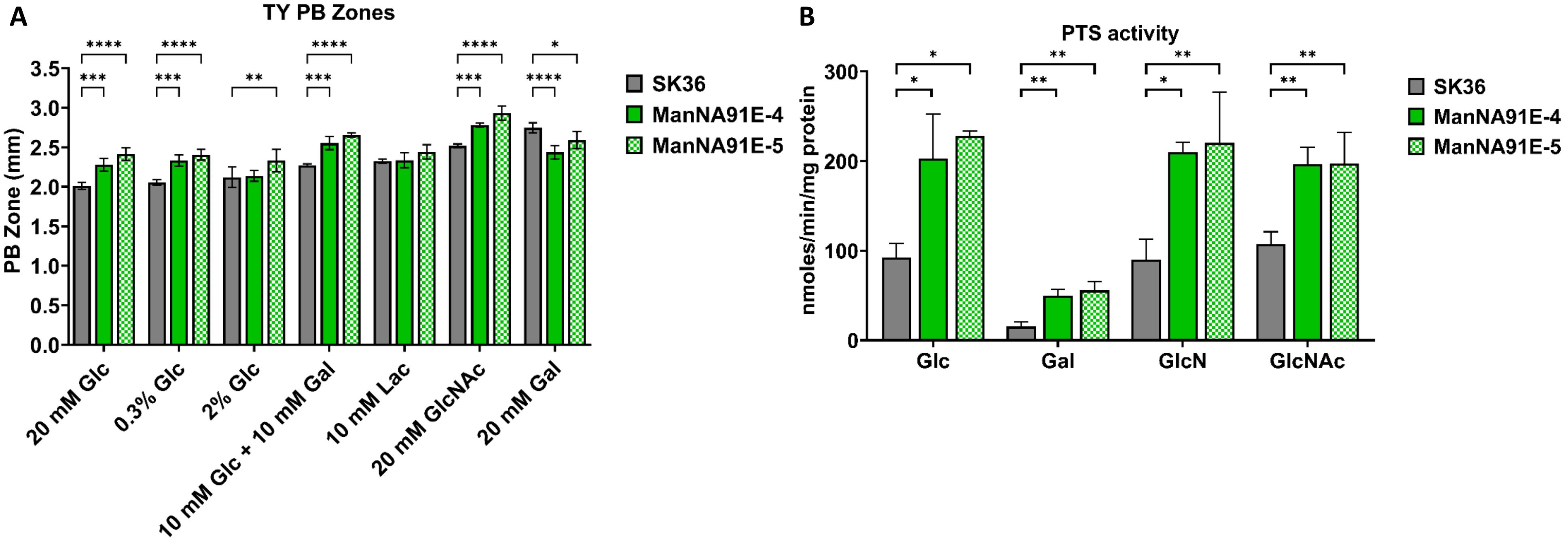

Fig S2 Characterization of two ManNA91E isolates. (A) For quantification of H_2O_2 excretion, 10 µl of cells were spotted onto TY agar plates supplemented with various sugars and incubated for 24 hours in a 5% CO_2 environment. Each PB zone was measured from the edge of the bacterial colony to the edge of the Prussian blue precipitation at four locations using ImageJ software. (B) To measure PTS activity, SK36 and ManNA91E cells were harvested from mid-exponential phase and subjected to an *in vitro* sugar phosphorylation assay (measuring oxidation of NADH). Results are each an average of at least three biological replicates. Asterisks represent statistical significance compared to the wild type according to two-way (A) or one-way (B) ANOVA (*, P < 0.05; **, P < 0.01; ***, P < 0.001; ****, P < 0.0001).

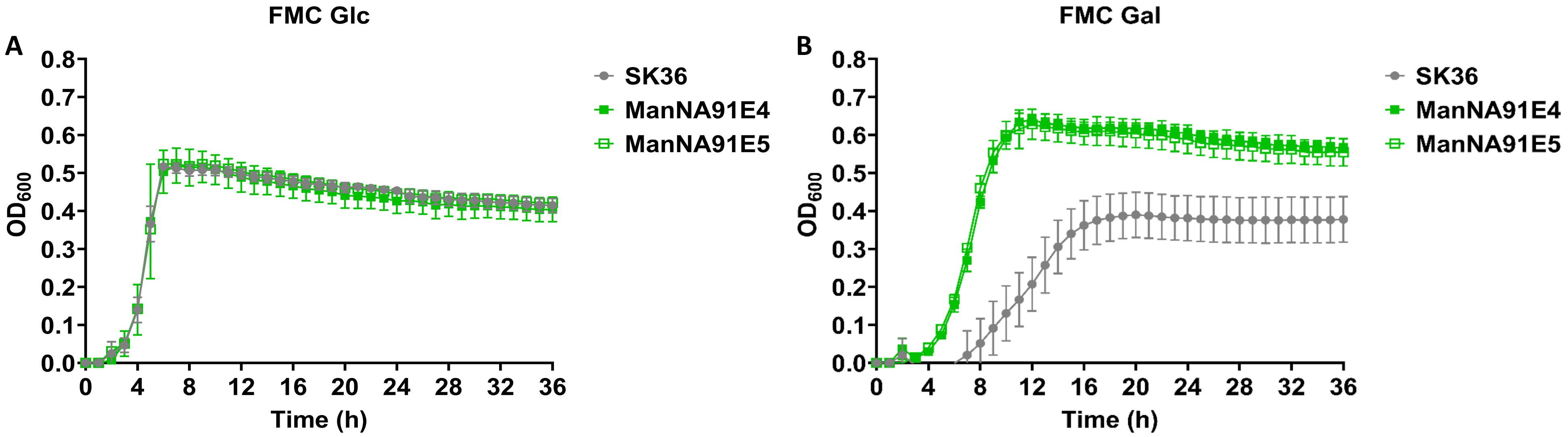

Fig S3 Characterization of two ManNA91E isolates. To measure growth, Wild type strain SK36 and two ManNA91E isolates were first cultured to mid-exponential phase in BHI and then diluted 1:100 into FMC medium supplemented with 20 mM glucose (A) or 20 mM galactose (B). Results are each an average of at least three biological replicates, with error bars denoting standard deviations. Optical density at 600 nm (OD_{600}) was monitored using a Bioscreen C over the course of 36 hours.

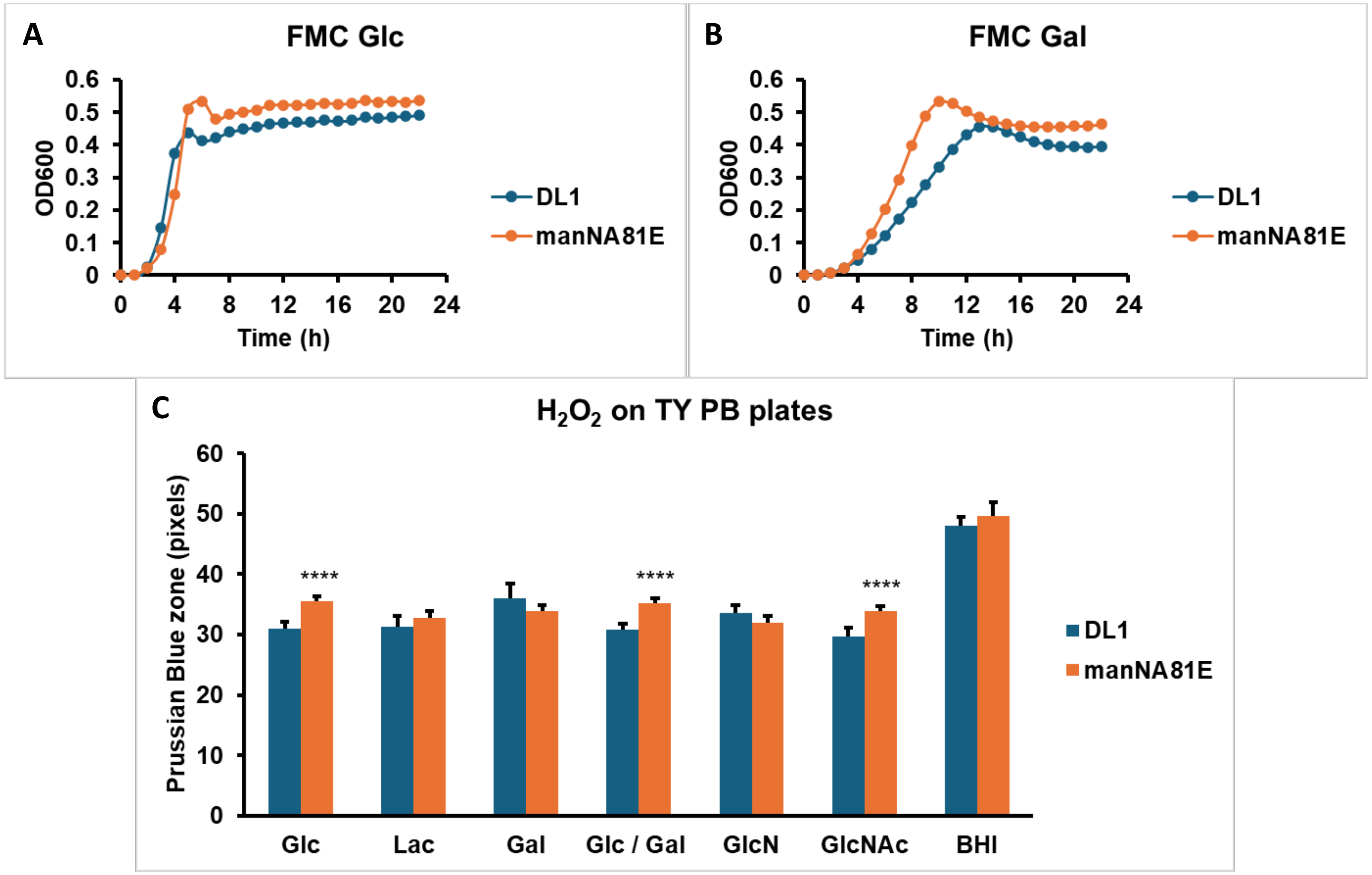

Fig S4 Characterization of *S. gordonii* and a manNA81E SNP mutant. (A-B) To measure growth, wild type strain DL1 and manNA81E were first cultured to mid-exponential phase in BHI and then diluted 1:100 into FMC medium supplemented with 20 mM glucose (A) or 20 mM galactose (B). Optical density at 600 nm (OD_{600}) was monitored using a Bioscreen C over the course of 36 hours. (C) For quantification of H_2O_2 excretion, 10 µl of cells were spotted onto TY agar plates supplemented with various sugars and incubated for 24 hours in a 5% CO_2 environment. Each PB zone was measured from the edge of the bacterial colony to the edge of the Prussian blue precipitation at four locations using Imager software. Results are each an average of at least three biological replicates, with error bars denoting standard deviations. Asterisks represent statistical significance compared to the wild type according to a students' T-test (C) (*, P < 0.05; **, P < 0.01; ***, P < 0.001; ****, P < 0.0001).

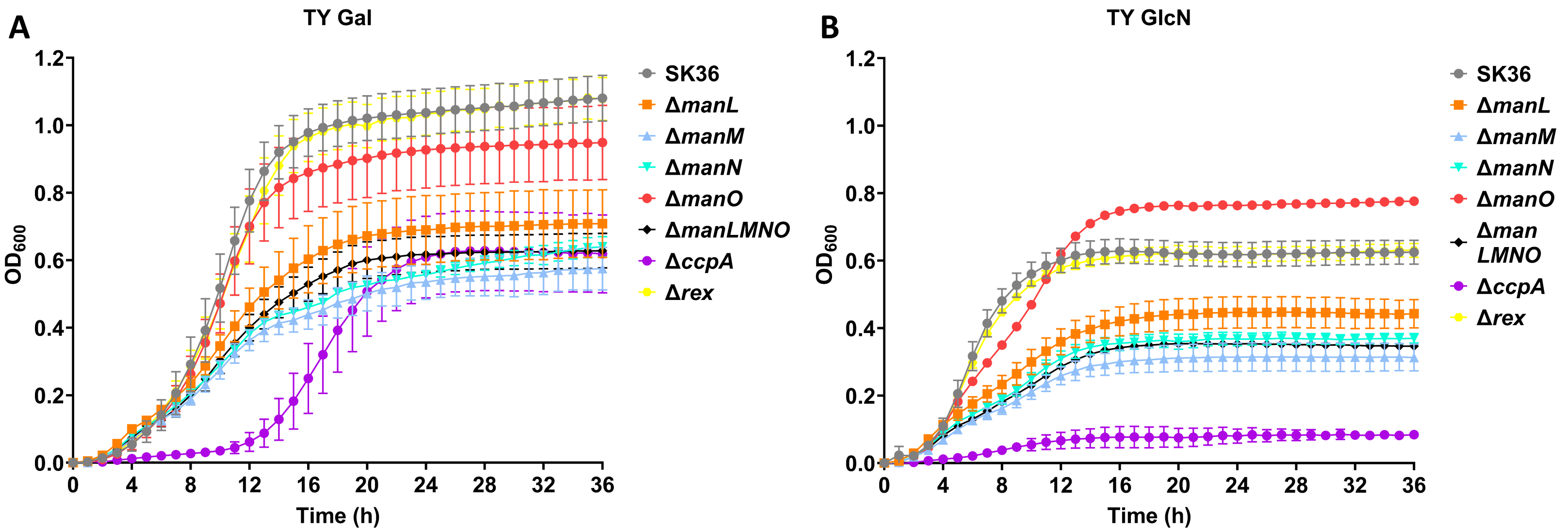

Fig S5 Growth curves of SK36 and glucose-PTS mutant derivatives. Wild type strains SK36 and its glucose-PTS mutants deficient in various PTS subunits were first cultured to exponential phase in BHI and then diluted 1:100 into fresh TY medium containing 20 mM of galactose (A), glucosamine (GlcN) (B), or N-Acetylglucosamine (GlcNAc) (C). Optical density at 600 nm (OD_{600}) was monitored using a Bioscreen C over the course of 48 hours. Results are each an average of at least four biological replicates.

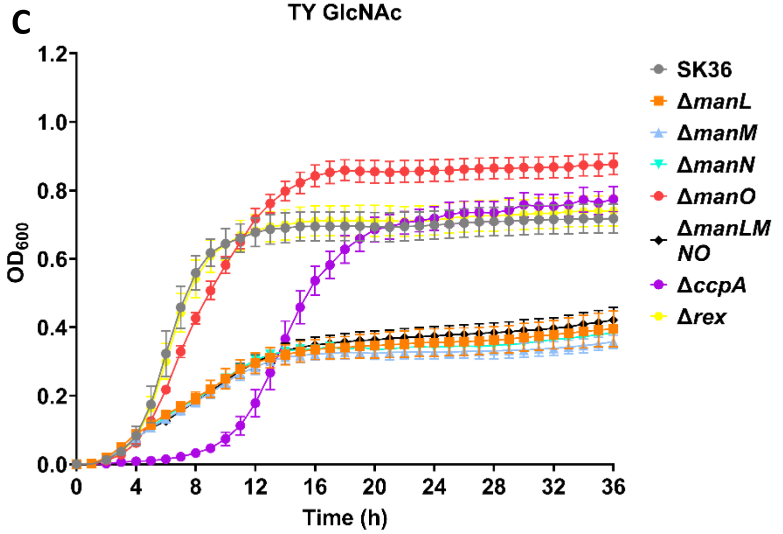

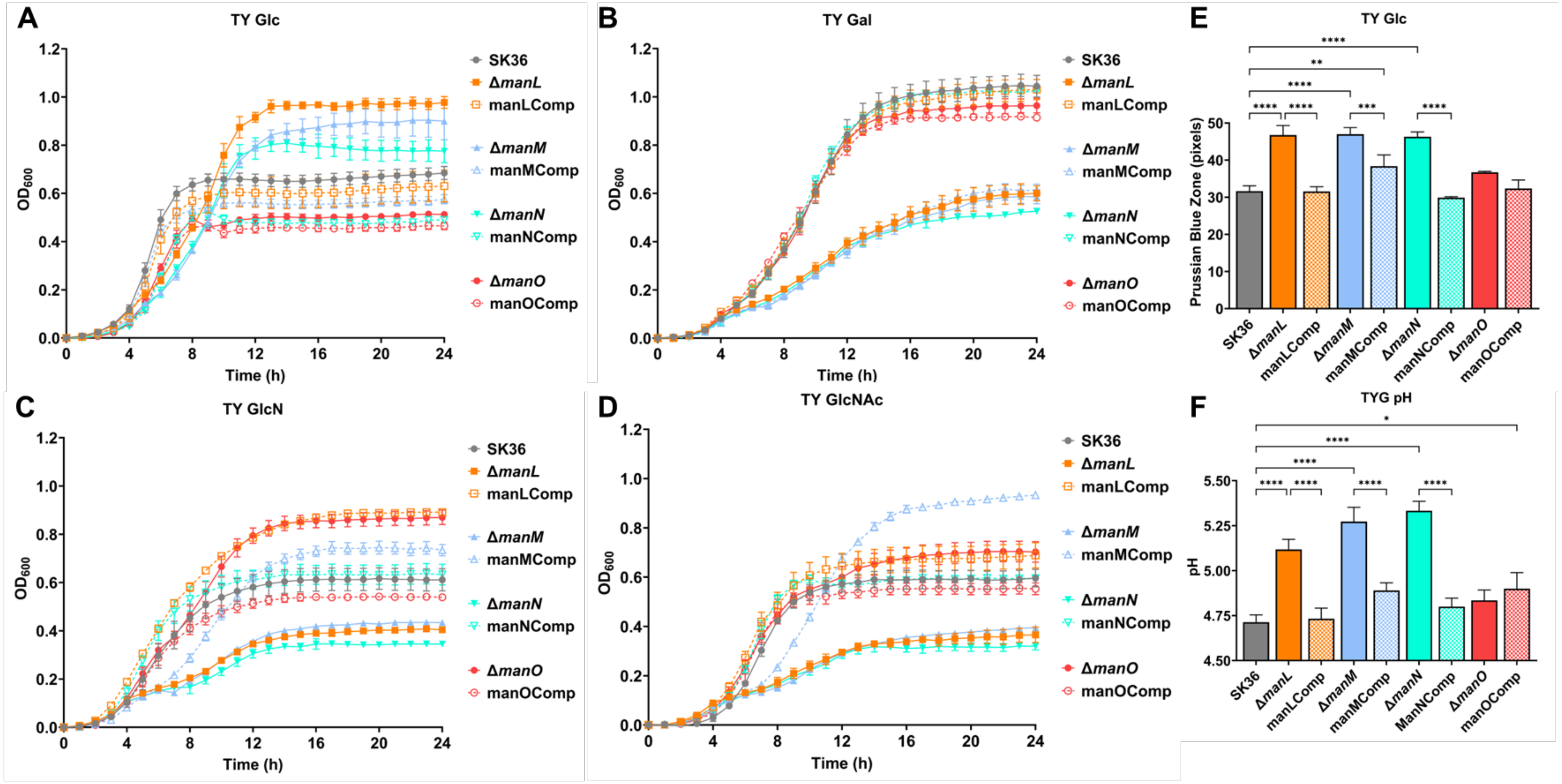

Fig S6 Phenotypic characterization of PTS complement mutants. To measure growth, SK36 and various mutants were first cultured to exponential phase in BHI and then diluted 1:100 into fresh TY medium containing 20 mM of glucose (A), galactose (B), glucosamine (C), or N-acetylglucosamine (D). Optical density at 600 nm (OD_{600}) was monitored using a Bioscreen C over the course of 24 hours. For quantification of H_2O_2 excretion, 10 µl of cells were spotted onto TY agar plates supplemented with glucose and incubated for 24 hours in a 5%-CO_2 environment. Each PB zone was measured from the edge of the bacterial colony to the edge of the PB precipitation at four locations using ImageJ software (E). To measure pH, cells were grown in TY supplemented with 20 mM glucose for 20 hours for pH measurement (F). Results are each an average of at least three biological replicates, with error bars denoting standard deviations. Asterisks represent statistical significance compared to the wild type, or respective complement, one-way ANOVA followed by Tukey's multiple comparisons test (E-F) (*, $P < 0.05$; **, $P < 0.01$; ***, $P < 0.001$; **** , $P < 0.0001$).

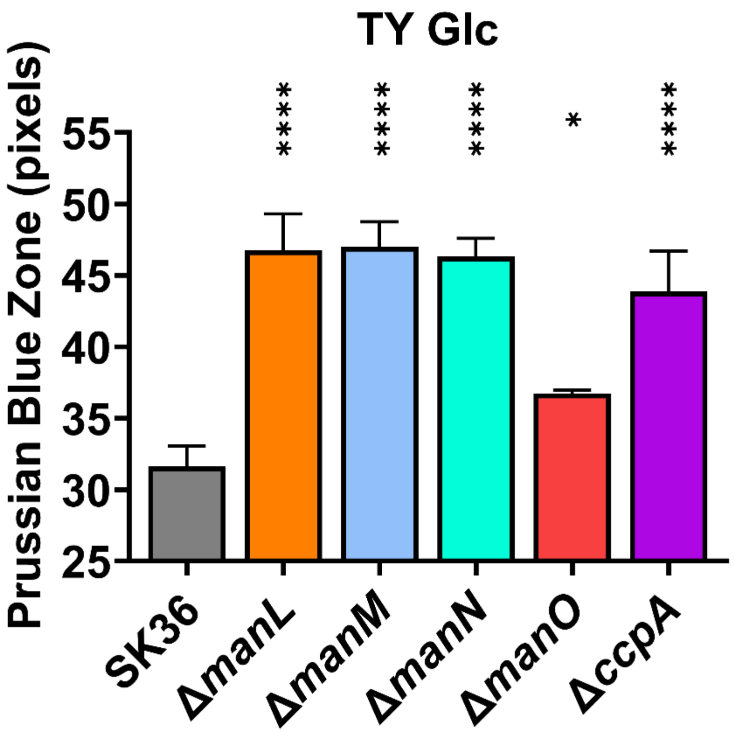

Fig S7 H_2O_2 secretion on TYG agar plates. For quantification of H_2O_2 excretion, 10 µl of cells were spotted onto TY agar plates supplemented with 20 mM glucose and incubated for 24 hours in a 5% CO_2 environment. Each PB zone was measured from the edge of the bacterial colony to the edge of the Prussian blue precipitation at four locations using ImageJ software. Results are each an average of at least three biological replicates, with error bars denoting standard deviations. Asterisks represent statistical significance compared to the wild type according to one-way ANOVA (*, P < 0.05; **, P < 0.01; ***, P < 0.001; ****, P < 0.0001).

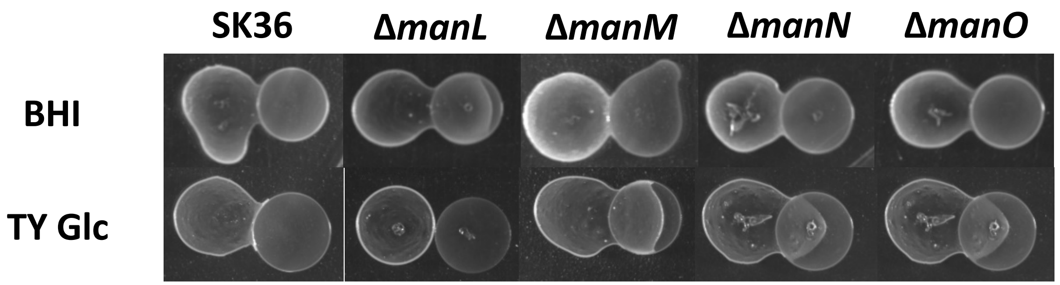

Fig S8 Antagonism of *S. mutans*. Cultures of SK36 and its mutant derivatives were each dropped onto the surface of either BHI or TY-agar plates supplemented with glucose and incubated for 24 hours in an aerobic environment supplemented with 5% CO_2. Cultures of *S. mutans* were then spotted to the left of the initial colonies with 50 µg/ml of catalase to degrade any H_2O_2 and were incubated for an additional 24 hours. Each experiment was repeated three times, with a representative result being presented.

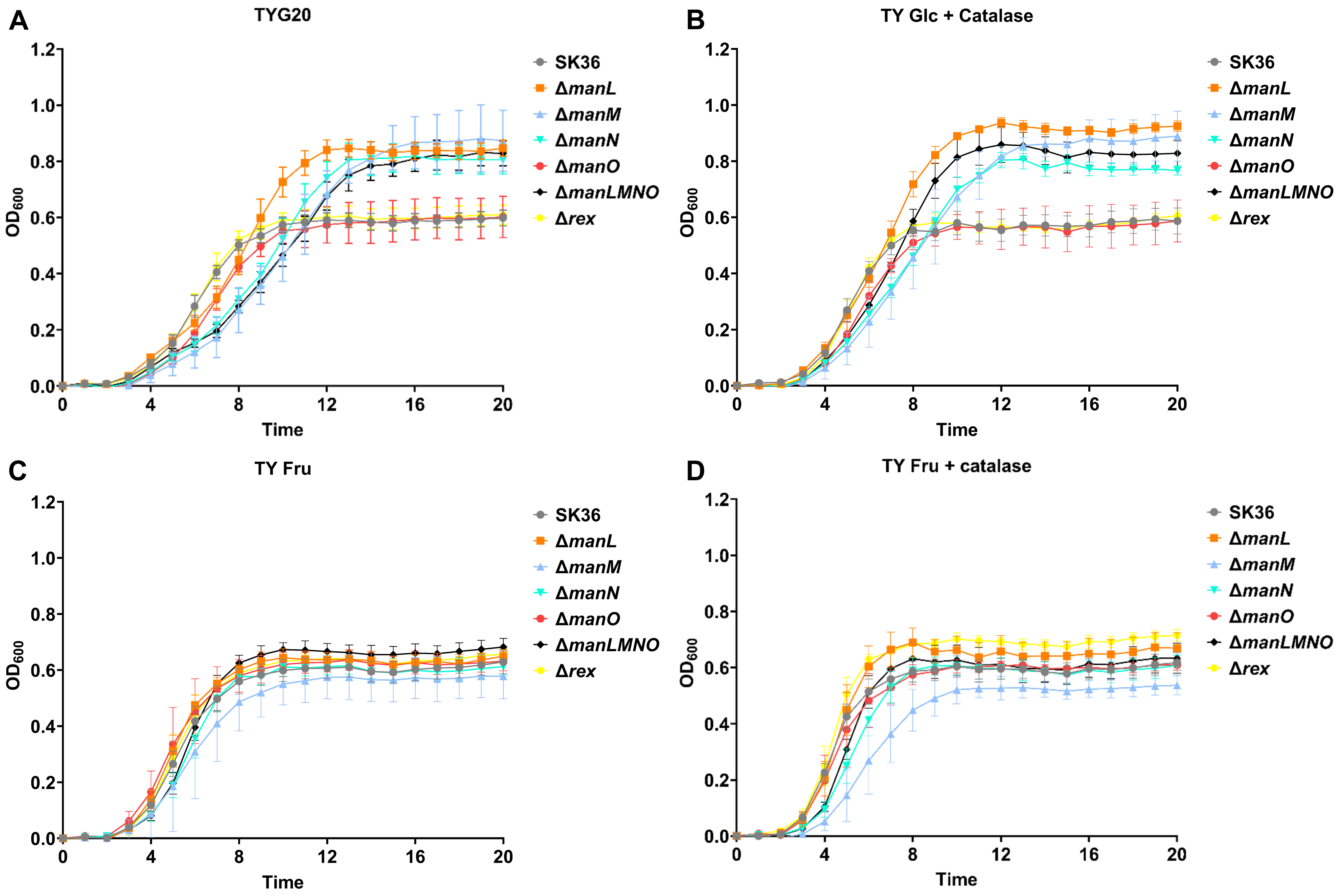

Fig S9 Growth curves of SK36 and its mutant derivatives with and without catalase. Wild type strains SK36 and its mutant derivatives were first cultured to mid-exponential phase in BHI and then diluted 1:100 into fresh TY medium containing 20 mM of glucose (A, B) or fructose (C, D). 50 μg/ml of catalase was added to degrade H_2O_2 produced by cells (B, D). Optical density at 600 nm (OD_{600}) was monitored using a Bioscreen C over the course of 20 hours. Results are each an average of at least four biological replicates.

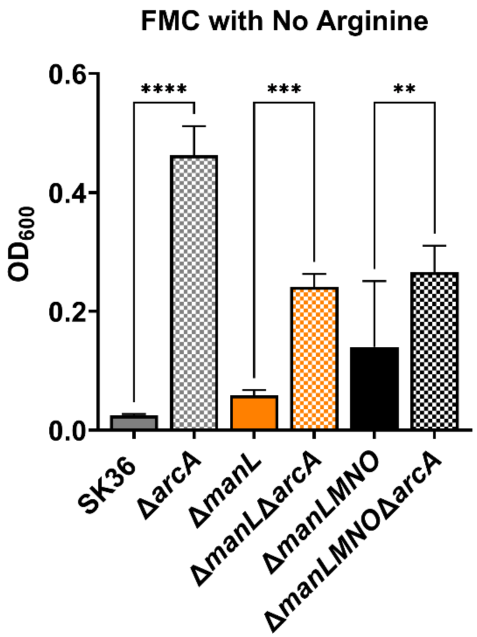

Fig S10 OD_{600} of FMC cultures prepared without arginine. SK36 and its mutant derivatives were grown overnight in BHI medium and diluted 1:100 into FMC without arginine, followed by 24 hours of incubation in an aerobic incubator maintained with 5% CO_2. Results are each an average of at least three biological replicates, with error bars denoting standard deviations. Asterisks represent statistical significance compared to the wild type according to Fishers LSD test (* , $P < 0.05$; ** , $P < 0.01$; *** , $P < 0.001$; **** , $P < 0.0001$).

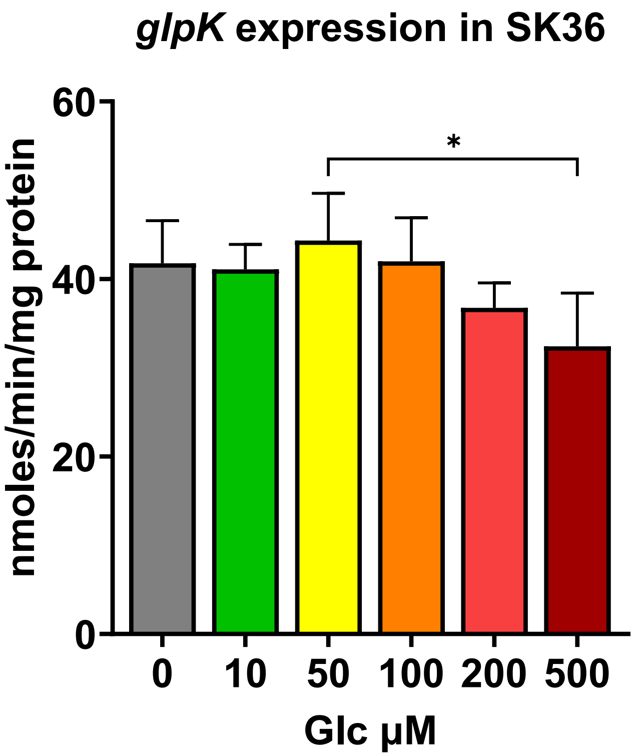

Fig S11 Downregulation of *glpK* gene expression in SK36 The activity of a *PglpK::cat* reporter fusion was measured in cells incubated in an aerobic environment supplemented with 5% CO_2 in TY medium supplemented with galactose to OD_{600} 0.3 and treated with increasing amounts of glucose for one hour. Cells were then harvested and subject to a chloramphenicol acetyltransferase activity assay. Results are the averages of three biological replicates, with error bars denoting standard deviations. Asterisks represent statistical significance compared to the wild type according to one-way ANOVA followed by Tukey's multiple comparisons test (*, $P < 0.05$; ** , $P < 0.01$; *** , $P < 0.001$; **** , $P < 0.0001$).

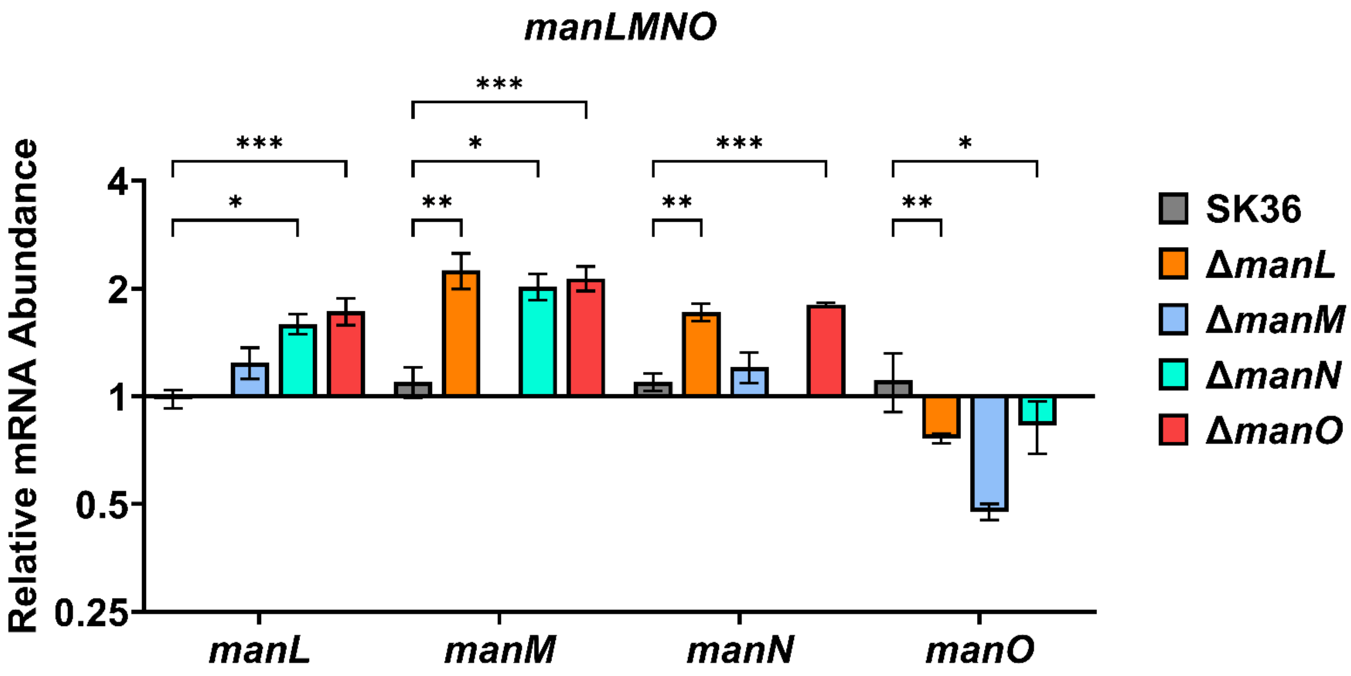

Fig S12 Transcription of the PTS operon by PTS deletion mutants. SK36 and its mutant derivatives were cultured in TY medium supplemented with 20 mM glucose to mid-exponential phase and RNA was extracted followed by reverse transcription and qPCR using an internal control (*gyrA*). Results are the averages of three biological replicates, with error bars denoting standard deviations. Asterisks represent statistical significance compared to the wild type according to two-way ANOVA followed by Tukey's multiple comparisons test (* , $P < 0.05$; ** , $P < 0.01$; *** , $P < 0.001$; **** , $P < 0.0001$).

Table S1. WGS analysis and variant calling of passaged cultures.

Position	Mutation	Annotation	Gene	Description
			**RD15-1**	
1,917,200	T→G	Silent	*mip*	Macrophage infectivity potentiator protein, putative
			**RD15-2**	
797,117	G→A	M1I (ATG→ATA)	SSA_0815	Hypothetical protein
1,884,389	G→A	E427L (GAA→AAA)	SSA_1885	Pseudogene
1,910,274	C→A	A91E (GCG→GAG)	*manN* →	Phosphotransferase system, mannose-specific EIID
			**RD15-3**	
685,547	+AA	Noncoding	SSA_0701	Cation transporter CorA family, putative

Table S2. WGS analysis and variant calling of ManNA91E isolates.

Position	Mutation	Annotation	Gene	Description
**ManNA91E-4**				
**304,427**	C→G	T434R (ACA→AGA)	*glnA* →	Glutamine synthetase type 1, putative
**972,376**	C→T	A275V (GCT→GTT)	SSA_0963 →	Peptidoglycan N-acetylglucosamine deacetylase A, putative
**1,063,922**	G→A	R279Q (CGG→CAG)	*potA* →	ABC transporter ATP-binding protein-spermidine/putrescine transport, putative
**1,910,274**	C→A	**A91E** (GCG→GAG)	SSA_1920 →	Phosphotransferase system, mannose-specific EIID, putative
**ManNA91E-5**				
**397,022**	G→A	V440V (GTC→GTT)	SSA_0400 ←	Conserved hypothetical protein
**445,960**	T→G	I178L (ATC→CTC)	SSA_0447 ←	Magnesium and cobalt transporter, putative
**510,435**	G→A	G229E (GGG→GAG)	*pduX* →	Propanediol utilization kinase, putative
**541,162**	C→G	D57E (GAC→GAG)	*aro* →	Phospho-2-dehydro-3-deoxyheptonate aldolase (DAHP synthatase), possibly tyr-sensitive, putative
**1,413,010**	G→A	D51D (GAC→GAT)	*pdxK* ←	Pyridoxal kinase, putative
**1,819,663**	C→A	G253W (GGG→TGG)	SSA_1829 ←	RNA methyltransferase, putative
**1,910,274**	C→A	**A91E** (GCG→GAG)	SSA_1920 →	Phosphotransferase system, mannose-specific EIID, putative

Table S3. SK36 and its mutant derivatives doubling time (T_d) and maximum OD after 48 hours of growth in TY media supplemented with various sugars. Asterisks represent statistical significance compared to the wild type according to Welch's T-test (*, $P < 0.05$; **, $P < 0.01$; ***, $P < 0.001$; ****, $P < 0.0001$).

Strain	TY Glucose		TY Galactose		TY Glucosamine		TY N-acetylglucosamine	
	T_d (min, avg ± SD)	Max OD_{600}	T_d (min, avg ± SD)	Max OD_{600}	T_d (min, avg ± SD)	Max OD_{600}	T_d (min, avg ± SD)	Max OD_{600}
**SK36**	76.11 ± 3.33	0.644 ± 0.05	116.12 ± 7.98	1.097 ± 0.07	87.68 ± 1.39	0.629 ± 0.04	78.25 ± 3.72	0.722 ± 0.04
**Δ*manL***	82.10 ± 4.69	1.012 ± 0.05 ****	215.13 ± 6.74 ****	0.714 ± 0.1 **	307.78 ± 9.79 ****	0.449 ± 0.04 ***	262.04 ± 15.54 ****	0.409 ± 0.05 ****
**Δ*manM***	109.60 ± 2.38 ****	0.98 ± 0.06 ****	248.56 ± 8.56 ****	0.585 ± 0.07 ***	310.57 ± 13.9 ****	0.336 ± 0.01 ****	252.26 ± 13.09 ****	0.395 ± 0.04 ****
**Δ*manN***	101.63 ± 3.96 ****	0.928 ± 0.09 **	248.85 ± 2.34 ****	0.665 ± 0.03 ***	282.41 ± 7.36 ****	0.377 ± 0.01 ****	238.88 ± 11.30 ****	0.416 ± 0.02 ****
**Δ*manO***	72.82 ± 1.95	0.633 ± 0.02	132.14 ± 4.06 *	0.960 ± 0.11	187.55 ± 5.41 ****	0.790 ± 0.01 ***	123.89 ± 4.12 ****	0.889 ± 0.03 ***
**Δ*manLMNO***	118.39 ± 6.87 ****	0.955 ± 0.12 **	229.77 ± 8.99 ****	0.632 ± 0.05 ***	321.18 ± 3.11 ****	0.355 ± 0.01 ****	237.12 ± 5.10 ****	0.455 ± 0.04 ***
**Δ*ccpA***	100.00 ± 2.44 ****	0.862 ± 0.03 ***	170.08 ± 9.11 ***	0.629 ± 0.12 **	-	0.091 ± 0.01 ****	126.66 ± 4.02 ****	0.789 ± 0.04
**Δ*rex***	74.18 ± 5.34	0.647 ± 0.02	130.02 ± 3.49*	1.103 ± 0.07	93.02 ± 6.12	0.636 ± 0.02	73.95 ± 3.48	0.746 ± 0.04

Table S4. Primers used in this study.

Primers	Sequence	Purpose
EIIAB_F	5’- GGA ATG TCA ATA ATG AGT ATC GGA ATC -3'	Sanger sequencing
EIIAB_R	5’- CTG CAC GTT GGC TTT CTT AAT C -3'	Sanger sequencing
EIIC_F	5’- GCC AAC GTG CAG TAG AAT CA -3'	Sanger sequencing
EIIC_R	5’- ACG CCA CCA AAC TTT TTG AC -3'	Sanger sequencing
EIID_F	5’- CCT AGA AGA CTA CTA AGA AAG GAG ACA -3'	Sanger sequencing
EIID_R	5’- GAA GGC CGA AAC CTT CTT TT -3'	Sanger sequencing
EIIGlcA_3GA_1	5’- ATA TTT TAC TGG ATG AAT TGT TTT AGT AGA AAG GAG GAA TGG AAG AAG CAA ATG CTC C -3'	Deleting EIIA
EIIGlcA-3GA_3	5’- AGT CAA ACA TTT TAT AAG GAG GAA TGC CTG CTG CGG CTG CT -3'	Deleting EIIA
EIIGlcA-3GA_2	5’- ATA TTT TAC TGG ATG AAT TGT TTT AGT AGA AAG GAG GAA TGG CTC CAG TTG CCC AAG CAG	Deleting EIIA
EIIGlcB-2GA	5’- GCC ATT TAT TAT TTC CTT CCT CTT TTA GGC AAG GTT GAT TTT GAG CTT -3'	Deleting EIIB
EIIGlcB-3GA	5’- ATA TTT TAC TGG ATG AAT TGT TTT AGT AGA GTA CTG TCA ATG GAC AAG GAA GA	Deleting EIIB
ManM2	5’- GCC ATT TAT TAT TTC CTT CCT TTA GGT TGG TGG AAT TGG -3'	Deleting *manM*
ManM3	5’- ATA TTT TAC TGG ATG AAT TGT TTT AGT AGA AAA GGC GGA AAT GGT GGT -3'	Deleting *manM*
ManN1	5’- TTG CTG GTC TCT TCT TGA CTA TG -3'	Deleting *manN*
ManN2	5’- GCC ATT TAT TAT TTC CTCT CTT TTA TCG TAG TTC CAA GAA CCT TGC -3'	Deleting *manN*
ManN3	5’- ATA TTT TAC TGG ATG AAT TGT TTT AGT AGA CTG CTT CTA ACT TTC GCT TGT ATG -3'	Deleting *manN*
ManO_F	5’- GGA GTT ACT CTA TGG CAC AGT C -3'	Sanger sequencing
ManO_R	5’- GGT TGA AAC TTC TTC AAC CCT AT -3'	Sanger sequencing
ManO2	5’- GCC ATT TAT TAT TTC CTT CCT CTT TTA CCC AAC CTT TCC GCC TAA T -3'	Deleting *manO*

ManO3	5’- ATA TTT TAC TGG ATG AAT TGT TTT AGT AGA CGG CAA TGA TAA GGT CGT TAG A -3'	Deleting *manO*
SK36_ptsH-1	5’- TTC TCT AAA TGT TTG AGT CCC ATG T -3’	Deleting *ptsH*
SK36_ptsH-2GA	5’- GCC ATT TAT TAT TTC CTT CCT CTT TTA GAA GCC ATA GTT AGA CTC TCC T -3’	Deleting *ptsH*
SK36_ptsH-3GA	5’- ATA TTT TAC TGG ATG AAT TGT TTT AGT AGA ACG ATG CTATCG CTG CAA TCT -3’	Deleting *ptsH*
SK36_ptsH-4	5’- CAA CAA CTG GTT TTC CAT TCA TGC CT -3’	Deleting *ptsH*
SSA_ptsH-S	5’- CCA GCA ACT TTG TTG GTT CA -3’	Deleting *ptsH*
SSA_ptsH-AS	5’- GGC CAA CAC CGA GAC TCA TA -3’	Deleting *ptsH*
Ssa_ccpA-1	5’- GCG GTC TAC TTC ATG AGC T -3’	Deleting *ccpA*
Ssa_ccpA-2GA	5’- GCC ATT TAT TAT TTC CTT CCT CTT TTA CTG TGT CGT CTG TGT TCA TAT AGT -3’	Deleting *ccpA*
Ssa_ccpA-3GA	5’- ATA TTT TAC TGG ATG AAT TGT TTT AGT AGA GCA TTA GCG AGC GTA AAT CAA CT -3’	Deleting *ccpA*
Ssa_ccpA-4	5’- TCC TCA TAG GAC GGG AAA AAG AA -3’	Deleting *ccpA*
SK36_arcA-1	5’- ATC ATG ATT ACC AAG GAG CAT TAT CAA T -3’	Deleting *arcA*
SK36_arcA-2GA	5’- GCC ATT TAT TAT TTC CTT CCT CTT TTA CAG TAC ATC CTT AAT ATT AGAATA TTT TGA AT -3’	Deleting *arcA*
SK36_arcA-3GA	5’- ATA TTT TAC TGG ATG AAT TGT TTT AGT AGA GTC TAT GCC ATT TGA ACG TGA AGA -3’	Deleting *arcA*
SK36_arcA-1	5’- CTG CTT GGT CAA AAT GAC GAG CA -3’	Deleting *arcA*
SSA_rex-1	5’- CGC TGA AAG CAA GGT TGG A -3’	Deleting *rex*
SSA_rex-2GA	5’- GCC ATT TAT TAT TTC CTT CCT CTT TTA CCT TTT AGC AGT AGC TCG AGG AA -3’	Deleting *rex*
SSA_rex-3GA	5’- ATA TTT TAC TGG ATG AAT TGT TTT AGT AGA GAC CTG ACT AGT GAA CTG CAG A -3’	Deleting *rex*
SSA_rex-4	5’- GTA GAA CAG AAG TTT ATC TAG GAA TAG AGG TT -3’	Deleting *rex*
ManO4	5’- GCA TGG AGG ATG TGG CAA TA -3'	Deleting *manO*
SSA_1918_2GA	5’- GCC ATT TAT TAT TTC CTT CCT CTT TTA CCG ATA CTC ATT ATT GAC ATT CCT C -3'	Deleting *manL*

SSA_1918_3GA	5'- ATA TTT TAC TGG ATG AAT TGT TTT ATT AGA GCC AAC GTG CAG TAG AAT CA -3'	Deleting *manL*
SK36_manL_Comp-2GA	5'- GCC ATT TAT TAT TTC CTT CCT CTT TTA TGA TTC TAC TGC ACG TTG GCT -3'	Complementing *manL*
SSA_manM-comp-2GA	5'- GCC ATT TAT TAT TTC CTT CCT CTT TTA TTC TTA GTA GTC TTC TAG GAT GTC -3'	Complementing *manM*
SSA_manN-comp-2GA	5'- GCC ATT TAT TAT TTC CTT CCT CTT TTA GAA GGC CGA AAC CTT CTT TTT G -3'	Complementing *manN*
SSA_manO-comp-2GA	5'- GCC ATT TAT TAT TTC CTT CCT CTT TTA GGT TGA AAC TTC TTC AAC CCT AT -3'	Complementing *manO*
SSA_EIIA_H13A_2	5'- CCA GCA GCA AAT TCA CCG GCG CTA GCA ATA ATG -3'	mutating SSA_manLH13A
SSA_EIIA_H13A_3	5'- GAA TCA TTA TTG CTA GCG CCG GTG AAT TTG CTG C -3'	mutating SSA_manLH13A
SSA_EIIB_H185A_2	5'- CCA AGC TGT TGC TAC CTG ACC GGC CAG CAA ACG TGT GTC G -3'	mutating SSA_manLH185A
SSA_EIIB_H185A_3	5'- CGA CAC ACG TTT GCT GGC CGG TCA GGT AGC AAC AGC -3'	mutating SSA_manLH185A
SK36_manL-S	5'- CCA ACG AAG GAC CAG ATG AT -3'	mutating SSA_manNA91E
Ssa_manN-AS	5'- AAGCAGTCCCATCAAACCAG -3'	mutating SSA_manNA91E
SSa-manNA91E-5'MAMA	5'- CCA ATC ATC GGA GTT ACA CTT CC -3'	mutating SSA_manNA91E
SGO_manNA81E-3	5'- GGG GGT TAC TCT CGA GCT TGA AGA AGA A -3'	mutating SGO_manNA81E
SGO_manNA81E_2	5'- GCG TTC TTC TTC AAG CTC GAG AGT AA -3'	mutating SGO_manNA81E
SGO_manN-4	5'- GCC AAT TGT TTG TAT CAA AGT AGG AA -3'	mutating SGO_manNA81E
SGO_manN-1	5'- GGT TTG GTA ACA GGT AAC TTA ACT G -3'	mutating SGO_manNA81E
SSA_Pglpk-5KpnI	5'- GCC CAG CAG GTA CCT ATC TAT TAT TT -3'	Mutation of *glpK*::CAT reporter
SSA_Pglpk-3Bm	5'- CTT GTG ACA TGG ATC CTC CTC CTA GTA AAA ATC TTG A -3'	Mutation of *glpK*::CAT reporter
GA-Km-5'	5'- TAA AAG AGG AAG GAA ATA ATA AAT GGC TAA -3'	Marker for GA
GA-Km-3'	5'- TCT ACT AAA ACA ATT CAT CCA GTA AAA TAT AAT ATT TT -3'	Marker for GA
GA-Em-5'	5'- TAA AAG AGG AAG GAA ATA ATA AAT GGC TAA CTA GAG GAT CTA GTC TTA TAA CTA TAC TG -3'	Marker for GA

GA-Em-3’	5’- TCT ACT AAA ACA ATT CAT CCA GTA AAA TAT GGA CGA CGA TGA CAA GTG AT -3’	Marker for GA
GA-Seq-5’	5’- TAG CCA TTT ATT ATT TCC TTC CTC TT -3’	Sanger sequencing
GA-Seq-3’	5’- ATA TTT TAC TGG ATG AAT TGT TTT AGT AGA -3’	Sanger sequencing
SSA_0737_S	5'- TGG CAA TAT CGT AGC TGC TG -3'	qRT-PCR
SSA_0737_AS	5'- TGT TGC GGT CAT ACA CCA CT-3'	qRT-PCR
SSA_0192_S	5' GAC CCA GCC ATC ATT CCT TA-3'	qRT-PCR
SSA_0192_AS	5'- AAG CAA GCC AGA TTC ACG AT -3'	qRT-PCR
SSA_1220_S	5'- AGC TGA TTG CCT TGA TTG CAG AC -3'	qRT-PCR
SSA_1220_AS	5'- ATC CGA AAA TTT ACG CTT GAC CT -3'	qRT-PCR
SSA_1221_S	5'- CTT CCT CGT TGC TGC TAA CC -3'	qRT-PCR
SSA_1221_AS	5'- GGA AGC GAG CTG AGT CAA GT -3'	qRT-PCR
SSA_0342_S	5'- CTT GGC AGG TCC TAC TGA GC -3'	qRT-PCR
SSA_0342_AS	5'- AGT GTC CAT TGG GAA ACG AG -3'	qRT-PCR
SSA_0391_S	5'- ATC ACT CAA CAC CGT CCA CTT CCA -3'	qRT-PCR
SSA_0391_AS	5'- TCT TCC AAG AAG AGG CGG AAT GGT -3'	qRT-PCR
SSA_1918_S	5’- CCA ACG AAG GAC CAG ATG AT -3’	qRT-PCR
SSA_1918_AS	5’- CAG CCA AGA CCA ACA CTT CA -3’	qRT-PCR
SSA_1919_S	5’- TGG GCT AAT ATC GGA GCT G -3’	qRT-PCR
SSA_1919_AS	5’- AGT ACG GAC AAT CAT AGT CAA G -3’	qRT-PCR
SSA_1920_S	5’- TTT GCC CAA GTA GGT CAA GG -3’	qRT-PCR
SSA_1920_AS	5’- AAG CAG TCC CAT CAA ACC AG -3’	qRT-PCR
SSA_1921_S	5’- GTT TAA TAC CAC TGG CGT ATC C -3’	qRT-PCR

SSA_1921_AS	5’- GCC AGA AAC ATT AGC ACC G -3’	qRT-PCR
